# Assessment of the Risk of Ebola Importation to Australia

**DOI:** 10.1371/currents.outbreaks.aa0375fd48a92c7c9422aa543a88711f

**Published:** 2014-12-10

**Authors:** Robert C. Cope, Phillip Cassey, Graeme J. Hugo, Joshua V. Ross

**Affiliations:** School of Mathematical Sciences, The University of Adelaide, Adelaide, South Australia, Australia; School of Earth & Environmental Sciences, The University of Adelaide, Adelaide, South Australia, Australia; Geography, Environment and Population, The University of Adelaide, Adelaide, South Australia, Australia; School of Mathematical Sciences & NHMRC CRE in Infectious Diseases Modelling to Inform Public Health Policy, Th University of Adelaide, Adelaide, South Australia, Australia

**Keywords:** Australia, ebola, Ebola virus, Policy, Risk

## Abstract

Objectives: To assess the risk of Ebola importation to Australia during the first six months of 2015, based upon the current outbreak in West Africa. Methodology: We assessed the risk under two distinct scenarios: (i) assuming that significant numbers of cases of Ebola remain confined to Guinea, Liberia and Sierra Leone, and using historic passenger arrival data into Australia; and, (ii) assuming potential secondary spread based upon international flight data. A model appropriate to each scenario is developed, and parameterised using passenger arrival card or international flight data, and World Health Organisation case data from West Africa. These models were constructed based on WHO Ebola outbreak data as at 17 October 2014 and 3 December 2014. An assessment of the risk under each scenario is reported. On 27 October 2014 the Australian Government announced a policy change, that visas from affected countries would be refused/cancelled, and the predicted effect of this policy change is reported. Results: The current probability of at least one case entering Australia by 1 July 2015, having travelled directly from West Africa with historic passenger arrival rates into Australia, is 0.34. Under the new Australian Government policy of restricting visas from affected countries (as of 27 October 2014), the probability of at least one case entering Australia by 1 July 2015 is reduced to 0.16. The probability of at least one case entering Australia by 1 July 2015 via an outbreak from a secondary source country is approximately 0.12. Conclusions: Our models suggest that if the transmission of Ebola remains unchanged, it is possible that a case will enter Australia within the first six months of 2015, either directly from West Africa (even when current visa restrictions are considered), or via secondary outbreaks elsewhere. Government and medical authorities should be prepared to respond to this eventuality. Control measures within West Africa over recent months have contributed to a reduction in projected risk of a case entering Australia. A significant further reduction of the rate at which Ebola is proliferating in West Africa, and control of the disease if and when it proliferates elsewhere, will continue to result in substantially lower risk of the disease entering Australia.

## Introduction

The largest outbreak of Ebola virus ever recorded continues to spread in West Africa. In response, the World Health Organisation (WHO) has declared a public health emergency of international concern. The potential for international dissemination of the Ebola virus, via international air travel, is an obvious risk that has already generated considerable interest^1^^,^ 2 ^,^ 3 ^,^ 4.

Preliminary assessments of the risk of international spread focused solely on the historic volume of international passenger flight traffic between countries 1 ^,^ 2. This has been followed by more detailed analyses, using historic passenger flight itinerary data to evaluate the expected number of internationally exported Ebola virus infections 4, and using a globally-connected metapopulation epidemic model that allows for epidemic outbreaks to be seeded via importation from passengers and then to dynamically evolve 3. Effectively, these latter two studies respectively consider the two distinct scenarios that we focus on herein: (i) assuming that significant numbers of cases of Ebola remain confined to Guinea, Liberia and Sierra Leone, and using historic Australian Customs passenger arrival card data into Australia; and, (ii) assuming potential global spread based upon historic international flight data.

On 27 October 2014 the Australian Government announced a policy change indicating that, effective immediately, new visas would not be granted, and existing temporary visas for individuals who had not yet departed to Australia would be cancelled 5. Similar measures have since been proposed in Canada 6. Visa restrictions, and in particular restrictions on humanitarian visas, pose a significant ethical challenge and have the potential for wide-ranging political ramifications. As such it is important to determine the efficacy of these policies in reducing the risk of Ebola arrival.

The relative risk to Australia, in comparison to countries such as Ghana, Senegal, and the United Kingdom, is small, and hence an assessment of the risk of Ebola importation to Australia has not been previously reported in existing studies, which where not focussed on any specific country. Such an assessment is of obvious benefit to decision makers within Australia, both in its own right, and to allow for the assessment of new visa restriction policy.

We develop data-informed models appropriate to each scenario, and parameterised these models using passenger arrival card or international flight data, and WHO case data from West Africa as at 3 December 2014 21 . An assessment of the risk under each scenario is reported. This consists of an estimate of the probability of importation at the beginning of each month between January 2015 and July 2015. This time period has been considered, as it is likely that wide-spread vaccination will not become available until April at the earliest 8, and hence the continued spread within West Africa is highly probable. In addition, we report a comparison between the predicted risk based on WHO case data reported on 17 October 2014 and 3 December 2014.

## Methods

***Direct travel model***

In order to assess the risk of an individual with Ebola travelling directly from West Africa to Australia, the direct travel model was constructed. Epidemic dynamics in each of Liberia, Sierra Leone, and Guinea were evolved via a discrete-time stochastic Susceptible-Exposed-Infectious-Removed (SEIR) 9 ^,^ 10 epidemic model (with timesteps of one day; details in Appendix 1), and then the risk of entry into Australia was calculated based on the number of individuals flying into Australia from each of these countries (i.e., irrespective of stopovers). All passenger data was compiled and provided by the Australian Department of Immigration and Border Protection (www.immi.gov.au). Australia is one of the few countries in the world which is able to accurately measure the number of people entering and leaving the nation 20. This arises from Australia’s global isolation and its island geography. With modern border surveillance systems this has meant that all movements into and out of the country are channelled through a relatively small number of sea and air ports, where there is an advanced electronic system to record their movements and some basic characteristics. These include: (i) their origin and destination; (ii) passenger status (i.e., Australian resident, tourist, or immigrant); and (iii) whether it is a permanent, long term (one year or more, but temporary) or short term movement.

The SEIR-type model is a standard epidemiological model for diseases with dynamics like those of Ebola, with the exposed period in particular necessary to account for the latency between initial exposure to the disease and the later onset of symptoms and infectiousness. More complex models have been used to analyse Ebola dynamics in some studies^3^^,^ 11, however SEIR provides sufficient detail in this context.

For a given day, the probability an exposed individual did not travel to Australia that day was:

\begin{equation*}q_t = \left(\frac{N_t - E_t}{N_t}\right)^{n_p},\end{equation*}

with \begin{equation*}\small{N_t}\end{equation*} the total number of individuals eligible to fly, \begin{equation*}\small{E_t}\end{equation*} the number of exposed individuals, \begin{equation*}\small{n_p}\end{equation*} the number of passengers arriving per day, and thus the cumulative probability that at least one exposed individual travelled to Australia on or before day T is:

\begin{equation*}p_T = 1 - \prod_{t\leq T} q_t.\end{equation*}

As a baseline case, we assumed a mean latent period of 5.3 days (i.e., σ = 1/5.3) and a mean infectious period of 5.61 days (i.e., γ = 1/5.61), based on parameters reported by Althaus 12. We estimated the contact rate β = 0.21 so as to ensure a resulting doubling time of approximately 45 days. This doubling time was calculated based on weekly new confirmed cases data for Sierra Leone 22. The average daily rate of passenger arrivals into Australia was calculated from each of Liberia, Sierra Leone and Guinea, both total arrivals and limited to solely Australian residents, from Australian Customs arrival data from 2004-05 to 2013-14.

We considered: a baseline scenario with these parameters and historical transport levels; a scenario in which transport from West Africa was reduced by 50%; a scenario under which visas from West Africa are cancelled and no longer granted (i.e., limiting entry to only Australian residents); and a scenario under which the Ebola contact rate within West Africa was reduced by 20%. We also considered model sensitivity to increases or decreases in mean latent period, in particular demonstrating the impact of increasing latent period to 10 days or decreasing to 3 days. We report median cumulative probabilities of a case entering Australia, based on 1000 simulation runs for each scenario, along with 95% prediction intervals in tables/figures.

All modelling and analysis was performed using R version 3.1.0^19^. Baseline model code is available at github.com/robert-cope/simEbola. We are unable to make specific flight or passenger data available at this time.

***Global network secondary outbreak model***

In order to assess the risk of an Ebola case entering Australia via an outbreak in a secondary source location (i.e., via an outbreak in a country that does not currently have an outbreak), the global network secondary outbreak model was constructed. Each country worldwide was treated as an individual population, connected through the global flight network. Within each country, spread of ebola was modelled via the same discrete-time stochastic SEIR epidemic model as in the previous section. Each day, the number of individuals in each class was updated, and individuals were allowed to fly between countries: the number of flying individuals between each country being the average daily number of flying individuals between each pair of airports in the countries in question. Data on the annual number of international flights per airport and the number of seats, per airplane per airport, travelling worldwide for the year 2013, were obtained from OAG Aviation Worldwide Ltd (www.oag.com/). Susceptible and exposed individuals (i.e., those either not infected or infected and not yet showing symptoms) were allowed to fly, and the number of exposed individuals flying was modelled as a binomial random variable with probability being the proportion of exposed individuals of those eligible to fly.

Simulations of this model were progressed 211 days (3 December 2014 -- 1 July 2015) and the spread and growth of Ebola virus cases into each country recorded. Disease parameters were as described above. We report results of: (i) a baseline model with historical infection and transport rates and uniform infection rates in each country; (ii) a scenario under which countries that have experienced at least 100 cases then have 50% reduced outgoing traffic; and (iii) a scenario in which higher economic status countries have reduced contact rate. We report, for each scenario, the cumulative probability of entry into Australia at each timestep based on 50 simulations, i.e., the proportion of those simulations for which an entry into Australia had occurred.

Specifically, for the economically-moderated contact rate scenario, countries were classified into four classes based on existing World Bank income classifications 16: low income, low-mid income, mid-high income, and high income. The contact rate within each country was modified based on this classification: low income countries used an unmodified contact rate; high income countries used a decreased contact rate, such that in these countries the resulting epidemic had \begin{equation*}\small{R_0 = 1}\end{equation*} and thus would not experience unmitigated growth; and low-mid and mid-high income countries were assigned contact rates equidistant between these two extremes.

***Time-series comparison***

These models were initially constructed based on WHO case data reported on 17 October 2014, and projected forward 200 days. New data became available while the study was in review, and results were subsequently updated to reflect these more recent data, as reported at 3 December 2014. Initial projections from 17 October data were based on a doubling time of 30 days, a conservative choice given the range of doubling times reported at the time^13^^,^ 14 ^,^ 15. Comparisons were made between projected risk into Australia based on this initial analysis (17 October) and the updated analysis (3 December).

## Results

***Direct travel model***

Under the baseline scenario of unchanged epidemic conditions and traffic from West Africa to Australia, the probability of a case entering Australia by 1 July 2015 is 0.34 (Figure 1, Figure 2). Under the scenario of 50% reduced traffic, the probability of a case by 1 July 2015 falls to 0.19 (Figure 3, Figure 4). New Australian Government policy, restricting/cancelling visas from West Africa into Australia, reduced the risk of entry to a probability of 0.16 by 1July 2015 (Figure 2, Figure 3).

**Direct travel model:**
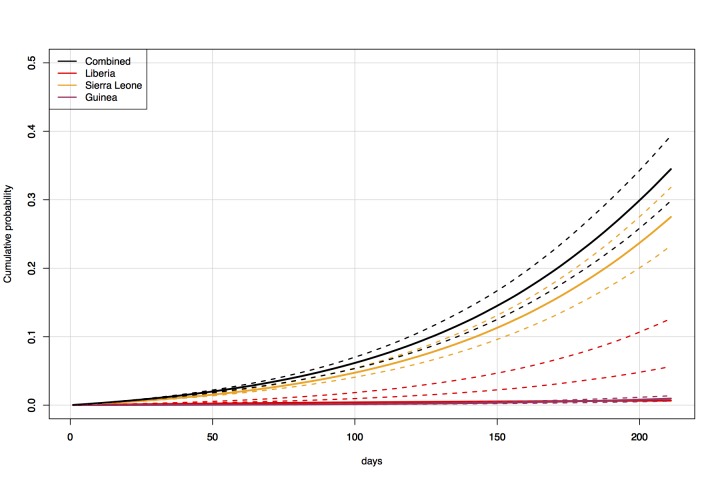
Cumulative probability over time (3 December 2014 — 1 July 2015) of an exposed individual flying into Australia from Liberia, Sierra Leone, and Guinea, with historic travel levels.

**Direct travel model:**
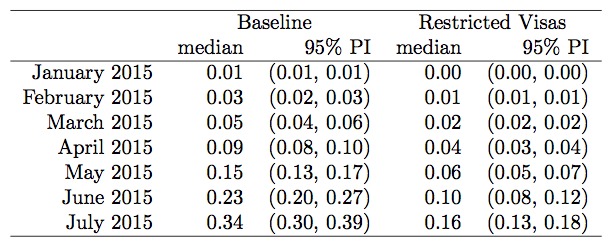
Probability of an exposed individual having entered Australia by the start of each month, based on historical travel levels (baseline) and new Australian Government visa restrictions from West Africa.

**Direct travel model:**
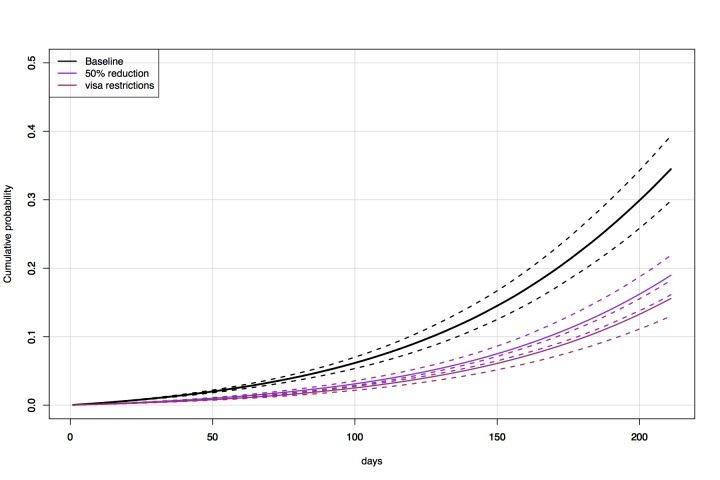
Cumulative probability over time (3 December 2014 — 1 July 2015) of an exposed individual flying into Australia for baseline, 50% flight reduction, and after visa restrictions.

**Direct travel model:**
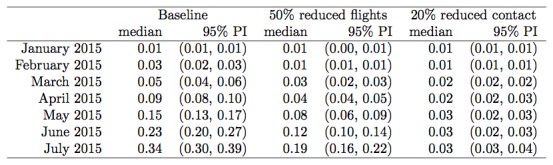
Probability of an exposed individual having entered Australia by the start of each month, based on historical travel levels (baseline), 50% reduced flights, or 20% lower contact rates within West Africa.

Alternately, when we consider the potential impact of reduced Ebola contact rates within existing outbreaks, a reduction of 20% results in a substantial reduction in risk, with the probability of a case entering by 1 July 2015 being only 0.03 (Figure 4, Figure 5).

**Direct travel model:**
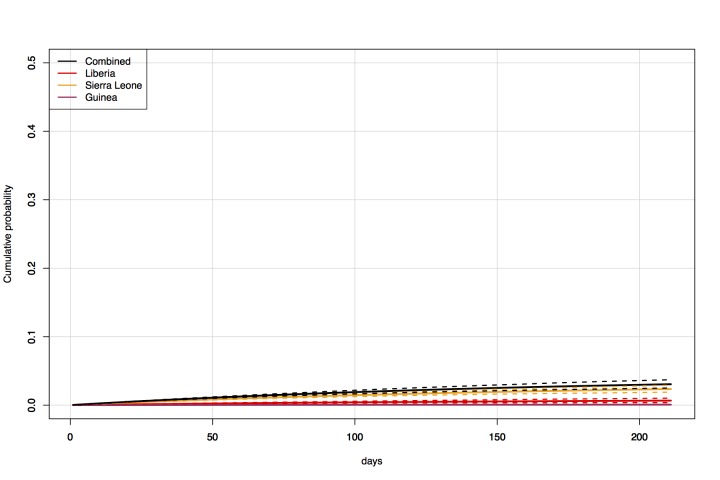
Cumulative probability over time (3 December 2014 — 1 July 2015) of an exposed individual flying into Australia from Liberia, Sierra Leone, and Guinea, after a 20% reduction in contact rate within these countries.

Increasing the latent period for Ebola to 10 days (provided the doubling time remains constant) increased the probability that a case enters Australia within a given time (Figure 6, Figure 7). The converse is also true - a decrease in latent period to 3 days decreased the probability of entry (Figure 6, Figure 7).

**Direct travel model:**
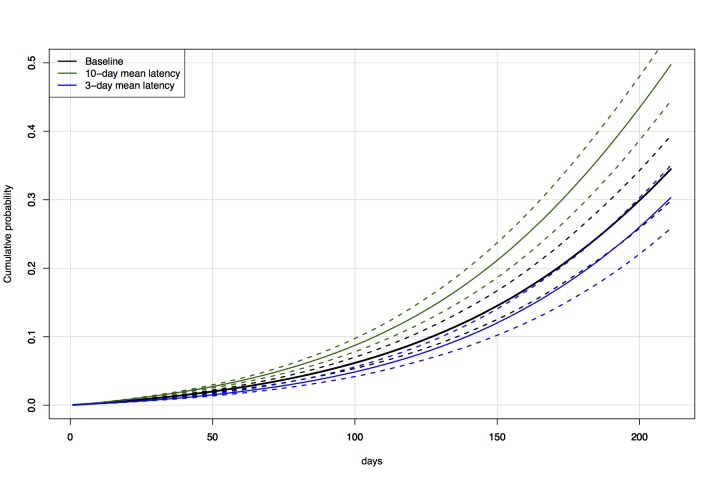
Cumulative probability over time (3 December 2014 — 1 July 2015) of an exposed individual flying into Australia, with variable mean latent period: baseline 5.3 days vs. 3 and 10 days.

**Direct travel model:**
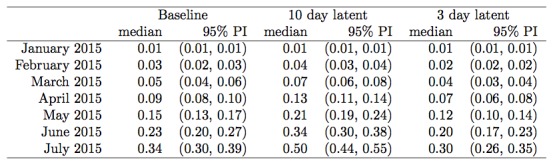
Probability of an exposed individual having entered Australia by the start of each month, based on historical travel levels and mean latent periods of 5.3 (baseline), 10, and 5 days.

***Global network secondary outbreak model***

Under a global outbreak model, with baseline parameters unchanged (infection rates globally uniform, consistent international air traffic), and based on 50 simulation runs, the first date a case entered Australia via an outbreak in a secondary source location was 23 May 2015, and cases had entered Australia by 1 June 2015 in 6% of simulation runs and by 1 July 2015 in 12% of simulation runs (Figure 8).

**Global network secondary outbreak model:**
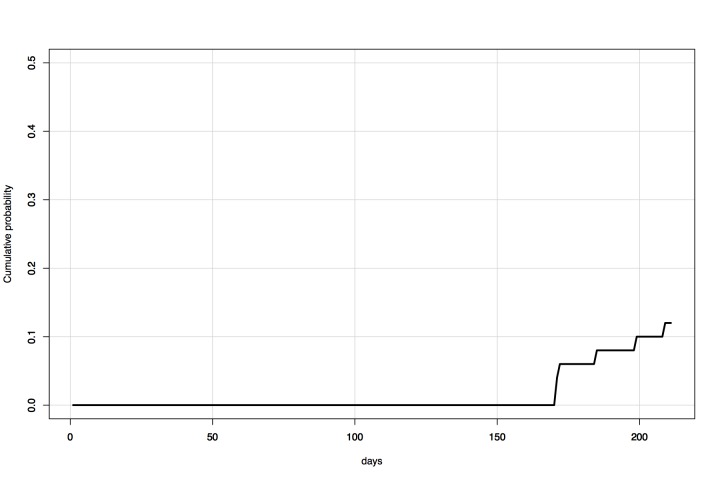
Cumulative probability over time of an exposed individual having entered Australia via an outbreak in a secondary source location. Based on 50 simulation runs.

Simulations were also performed under two alternate scenarios: (a) the rate of air traffic leaving infected countries was decreased by 50% for each country that has experienced at least 100 cases, and (b) contact rates were decreased within higher-income countries. Under both of these scenarios, no Ebola cases entered Australia by 1 July 2015 under 50 simulations of the global network secondary outbreak model.

***Time-series comparison***

Under historic traffic levels from West Africa to Australia (i.e., the direct travel model), and epidemic parameters and initial conditions as reported on 17 October 2014, the probability of a case entering Australia by 1 April 2015 was 0.97. The predicted risk under the same model, with parameters and initial conditions as reported on 3 December 2014, was 0.09 (Figure 9). The probability of a case entering within 200 days of 17 October 2014 was 1.00, compared to a probability of 0.30 within 200 days of 3 December 2014.

**Time-series comparison:**
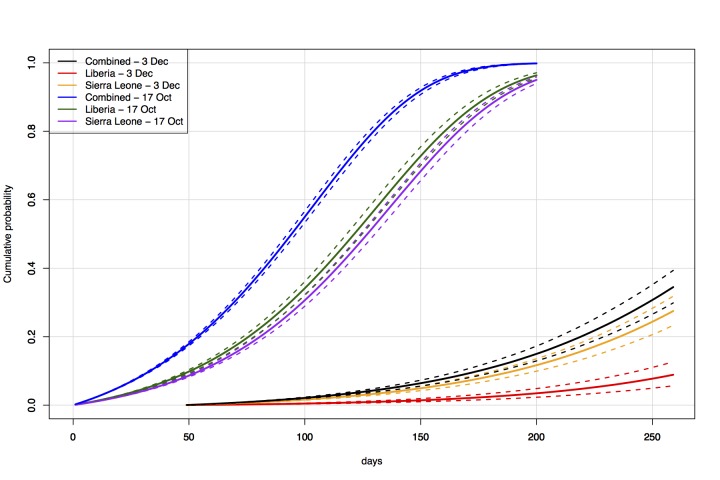
Projected risk over time (17 October 2014 -- 1 July 2015) of entry of a case of Ebola into Australia via direct travel from West Africa. Presents results of models based on data available at and initialized on each of 17 October 2014 and 3 December 2014.

Under the Global network secondary outbreak model, the probability of a case entering Australia via an outbreak in a secondary source location within 200 days of 17 October 2014 was 0.76. With updated parameters and initial conditions, the probability of a case entering Australia within 200 days of 3 December 2014 was 0.10 (Figure 10).

**Time-series comparison:**
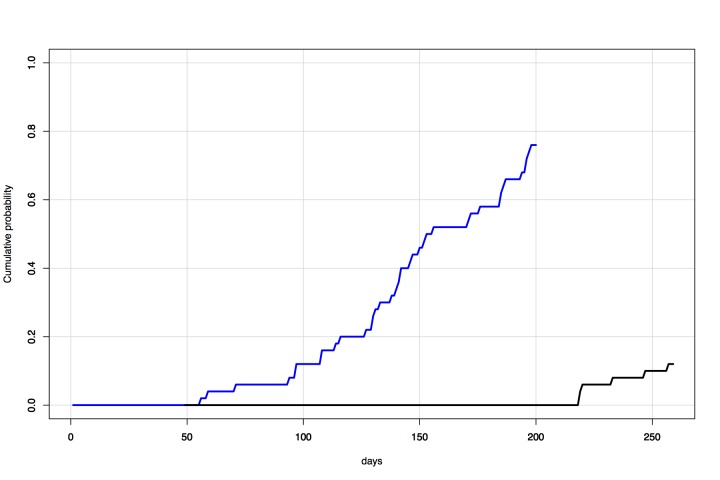
Projected risk over time (17 October 2014 -- 1 July 2015) of entry of a case of Ebola into Australia via an outbreak in a secondary source location. Presents results of 50 simulations each based on data available at and initialized on each of 17 October 2014 (blue) and 3 December 2014 (black).

## Discussion

***Direct travel model***

Under current epidemic conditions and historic travel levels into Australia, it is possible that an Ebola case will enter Australia within the first six months of 2015, having travelled directly from West Africa, with a probability of 0.34.

The cessation of granting visas/cancelling existing visas is effectively equivalent to a traffic reduction of approximately 60% (i.e., 83% reduction from Guinea, 60% reduction from Liberia, 56% reduction from Sierra Leone), and its impact is in line with this: the probability of a case entering Australia by 1 July 2015 is reduced by 53% (slightly more than under the 50% reduction in traffic scenario). However, the probability of an eventual case entering Australia within the first six months of 2015 is still sufficiently high as to warrant caution (16%).

It is possible that there may be some decrease in the number of Australian residents travelling to and from affected countries, which may further decrease the probability of a case arriving. Alternatively there may be, within the short term, an increase, if for example visitors to West Africa are returning to Australia at a greater rate than they may previously have in an attempt to avoid Ebola.

A 20% decrease in contact rate within affected West African countries reduced the probability of an eventual case entering Australia substantially (3% chance of introduction by 1 July 2015, vs. 34% under the baseline scenario). It is possible that public health research to determine effective ways to reduce infection rates, combined with foreign aid contributing to increased availability of hospital beds and high- quality treatment, could feasibly result in a decrease in contact rate of this magnitude. Note that at this level of reduced contact, the number of cases no longer increases exponentially, or, rather, the exponential growth is so slow that within the time period considered it is close to linear (Figure 5). If the contact rate is reduced even further than this, the number of Ebola cases will begin to decrease within West Africa. This is consistent with CDC predictions, that Ebola infection decreases under potential control and hospitalization scenarios 14.

***Global network secondary outbreak model***

We found that, under existing Ebola transmission parameters and historic global flight conditions, it is possible but not likely that Australia may see an Ebola case via an outbreak in a secondary source country within the first six months of 2015, with a probability of approximately 0.12 by 1 July 2015. It is very unlikely that this happens early during this time period, given the time it would take for outbreaks to be established in countries with significant direct air traffic to Australia.

Under a model with global control of air traffic leaving each country in which a significant outbreak has occurred, the probability of a case reaching Australia within the first six months of 2015 is further reduced, such that no simulation runs (from 50) had cases enter Australia within this interval. Some reduction in air traffic to and from affected countries is a reasonable assumption, either due to mandated restrictions, or just the natural desire of people to avoid travelling where epidemic risk is significant.

When the assumption is made that contact rates are likely to be reduced in higher-income countries, which may be reasonable due to a combination of high-quality healthcare, and education relating to disease transmission, global outbreak spread slows significantly. As a result of this, no simulation runs had a case enter Australia within the first six months of 2015 under this scenario.

It may appear unintuitive that there would be less risk of an Ebola case entering Australia within the first six months of 2015 from the global outbreak model than from direct travel. The discrepancy is due to the time scale involved: under the global outbreak model, secondary outbreaks would need to occur and grow in countries with direct connections to Australia for a case to then enter, which would take a significant amount of time. If the time scale were longer, the risk due to global spread would increase and eventually be greater than due to direct travel, and also be less susceptible to control measures such as visa restrictions.

***Time-series comparison***

Modelling based on updated parameters and initial conditions, based on data available at 3 December 2014 21, projected substantially lower risk of a case entering Australia than modelling based on parameters and initial conditions from 17 October 2014 7. For any given date, it is natural that risk under a model beginning in December would be lower than risk under a model beginning in October given that these probabilities are conditional on not having seen a case, i.e., projections from the October model include some risk that the case may have arrived in November. In addition to this, data available in December implied a slower doubling time of 45 days, whereas 17 October models relied on a doubling time of 30 days, chosen conservatively based on a variety of figures reported in the literature 13 ^,^ 14 ^,^ 15. Furthermore, initial case numbers based on 3 December data were lower, now taken from new weekly case counts consisting only of confirmed cases. Case numbers in Sierra Leone were higher than those in Liberia under 3 December data, resulting in greater greater risk of a case entering Australia due to direct travel from Sierra Leone, whereas under 17 October data Liberia presented more risk (Figure 8).

It is likely that the strong difference between results based on these two datasets is primarily due to two factors: (1) efforts to control the spread of Ebola in West Africa, and (2) more accurate data, restricted to confirmed cases. Significant public health measures for the control of Ebola are underway, and show promising signs. The number of new reported incidences in Liberia was stable or declining by 3 December 2014, and protocols were in place throughout the region to effectively isolate patients, and to ensure safe burial practices 21. These control measures would directly influence the rate at which the outbreak is growing, i.e., the doubling time. Data on new weekly cases, restricted to confirmed cases only, were not available when this analysis was performed on 17 October, and as a result the 3 December model uses these lower, more accurate initial estimates, which further slows outbreak growth and results in reduced projected risk to Australia. Overall, it is clear that there can be significant variability in estimated risk due to the parameter estimates used, and the reduction in risk projected here is likely due to both control efforts and improved data.

***Study assumptions and limitations***

The stochastic SEIR model used here effectively represents the necessary components of Ebola dynamics for this study. More complex models have been applied in other studies, incorporating e.g., specific hospitalisation dynamics or separate removal classes (death vs. recovery) allowing specific incorporation of post-death contact. However, in this study it was most parsimonious to use a simple model with fewer assumptions as to disease dynamics or model parameters. There is some variation in reported parameter values in the literature, e.g., in terms of reported latent period (12 vs. 13), and for the case of latent period we investigated a selection of values to determine sensitivity to this parameter.

One assumption made here, that is likely to significantly influence our predictions, is of consistency, i.e., the assumption that in general future disease dynamics and/or transport dynamics will follow past dynamics. If measures to control Ebola within West Africa are successful in the near future, or if air traffic trends from affected nations have been decreased significantly, then the risk of transport will be decreased. The best case scenario is of control within West Africa such that disease cases decrease to the point of eventual extinction without extensive outbreaks elsewhere (i.e., any individual cases that emerge elsewhere are controlled quickly). In a sense, the status-quo is the most conservative scenario.

We assumed here that 50% of removed individuals die, and 50% recover. Estimates of mortality rates for Ebola have varied considerably 13 ^,^ 15, and tend to change quickly within this outbreak, in part due to estimates being biased during the early stages of an outbreak 17. A higher mortality rate would, in this model, mean a faster increase in case numbers, and hence would lead to higher probabilities of introduction into Australia. As such, 50% is a conservative choice of mortality rate.

Overall, we have made a large number of assumptions in each of the alternate scenarios we have chosen. The extent to which air traffic, or disease contact rates might decrease is uncertain and will have a nontrivial impact on model results. In particular the choice of contact rate for countries within different economic groups essentially defines that model. In this case, we assumed that high income countries would have contact rates that result only in replacement, on average, in terms of outbreak growth (and as such outbreaks in these countries will die out via stochasticity). This seems reasonable, and is not inconsistent with high quality medical care, contact tracing, etc., but control could certainly be stronger or weaker than this.

Finally, it should be noted that these projections are based upon WHO infection numbers, which it has been suggested may be under-reporting significantly 14 ^,^ 18. If existing case numbers in West Africa are significantly higher than recorded, the disease would propagate more quickly and the probability of entry into Australia within a given timeframe would be higher.

***Conclusion***

Based on two alternate models for the spread of Ebola, either via direct travel from West Africa or through spread to secondary sources, we conclude that under existing conditions it is possible that a case of Ebola will enter Australia within the first six months of 2015, with a probability of entry of 0.34 by 1 July 2015 under the baseline direct travel scenario. Reduced traffic due to new government visa restrictions will decrease the probability of this occurring. Comparison between data from 17 October 2014 and 3 December 2014 suggests that control measures within this period have had a positive impact, resulting in reduced risk of importation into Australia. Further control of existing outbreaks within West Africa, and in any further outbreaks in secondary locations, would provide the strongest decrease in risk to Australia. Medical professionals and policy makers should be prepared for the possible entry of an Ebola case into Australia, and continue to undertake public health research and supply aid in an effort to effectively reduce proliferation of Ebola in existing outbreaks.

## Appendix 1

### SEIR Model

The stochastic SEIR model, for each country, is evolved forward over 200 daily timesteps.

Specifically, the transitions are:

\begin{equation*}S_{t+1} = \textrm{max}(0,\ S_t-d_t^1)\\\%0AE_{t+1} = \textrm{max}(0,\ E_t+d_t^1 - d_t^2)\\\%0AI_{t+1} = \textrm{max}(0,\ I_t+d_t^2 - d_t^3)\\\%0AR_{t+1} = R_t+d_t^3\end{equation*}

with:

\begin{equation*}d_t^1 \sim \textrm{Poisson}(\beta S_t I_t / (S_t + E_t + I_t + 0.5R_t))\\\%0Ad_t^2 \sim \textrm{Poisson}(\sigma E_t)\\\%0Ad_t^3 \sim \textrm{Poisson}(\gamma I_t)\end{equation*}

This formulation is similar in concept to that of the τ-leaping approximation for a continuous-time Markov chain. We take \begin{equation*}\small{0.5R_t}\end{equation*} base on the assumption of a case fatality proportion (rate) of 50%, i.e., that 50% of removed individuals are deaths and the remaining 50% are recovered and immune.

## References

[1] Brockmann, D., Schaade, L., Verbee, L. (2014) Ebola Outbreak. Worldwide Air-Transportation, Relative Import Risk and Most Probable Spreading Routes.

[2] Mekaru, S. (2014) Ebola 2014: A Rapid Threat Assessment. The Disease Daily.

[3] Gomes, M.F.C., Piontti, A.P., Rossi, L. et al. (2014) Assessing the International Spreading Risk Associated with the 2014 West Africa Ebola Outbreak. PLoS Curr. September 2. (doi:10.1371/currents.outbreaks.cd818f63d40e24aef769dda7df9e0da5). 10.1371/currents.outbreaks.cd818f63d40e24aef769dda7df9e0da5 PMC4169359 25642360

[4] Bogoch, I.I., Creatore, M.I., Cetron, M.S. et al. (2014) Assessment of the potential for international dissemination of Ebola virus via commercial air travel during the 2014 West African outbreak. The Lancet (doi:10.1016/S0140-6736(14)61828-6). 10.1016/S0140-6736(14)61828-6 PMC4286618 25458732

[5] Australian Broadcasting Corporation (2014) Federal Government to stop processing visa applications from countries affected by Ebola. Online news article; accessed 28 October 2014.

[6] Canadian Broadcasting Corporation (2014) Ebola: Canada suspending visas for residents of outbreak countries. Online news article; accessed 2 November 2014.

[7] World Health Organization (2014) WHO: Ebola Response Roadmap Update – 17 October 2014 Online data summary; accessed 21 October 2014.

[8] World Health Organization (2014) WHO High-level meeting on Ebola vaccines access and financ- ing. Summary Report. Accessed 29 October 2014.

[9] Keeling, M. & Rohani, P., (2008) Modeling infectious diseases in humans and animals. Princeton University Press.

[10] Anderson, R. & May, R., (1992) Infectious diseases of humans. Oxford University Press.

[11] Lewnard, J.A., Ndeffo Mbah, M.L., Alfaro-Murillo, J.A. et al. (2014) Dynamics and control of Ebola virus transmission in Monserrado, Liberia: a mathematical modelling analysis The Lancet Infectious Diseases (doi:10.1016/S1473-3099(14)70995-8). 10.1016/S1473-3099(14)70995-8 PMC4316822 25455986

[12] Althaus C.L. (2014) Estimating the Reproduction Number of Ebola Virus (EBOV) During the 2014 Outbreak in West Africa. PLOS Currents Outbreaks (doi:10.1371/currents.outbreaks.91afb5e0f279e7f29e7056095255b288). 10.1371/currents.outbreaks.91afb5e0f279e7f29e7056095255b288 PMC4169395 25642364

[13] WHO Ebola Response Team (2014) Ebola Virus Disease in West Africa – The First 9 Months of the Epidemic and Forward Projections. New England Journal of Medicine 371(16) pp 1481–1495. (doi:10.1056/NEJMoa1411100). 10.1056/NEJMoa1411100 PMC4235004 25244186

[14] Meltzer, M.I., Atkins, C.Y., Santibanez, S. et al. (2014) Estimating the future number of cases in the Ebola epidemic – Liberia and Sierra Leone, 2014–2015. MMWR 2014; 63 (Suppl-3). pp 1–14. 25254986

[15] Gire, S.K., Goba, A., Andersen, K.G. et al. (2014) Genomic surveillance elucidates Ebola virus origin and transmission during the 2014 outbreak. Science 345 pp 1369–1372. (doi:10.1126/science.1259657). 10.1126/science.1259657 PMC4431643 25214632

[16] World Bank Data: Country and Lending Groups. Accessed online 29 Oct 2014.

[17] Kucharski, A.J. and Edmunds, W.J. (2014) Case fatality rate for Ebola virus disease in west Africa. The Lancet (doi:10.1016/S0140-6736(14)61706-2). 10.1016/S0140-6736(14)61706-2 25260235

[18] World Health Organization (2014) WHO: Ebola Response Roadmap Situation Report – 15 October 2014 Online data summary; accessed 21 October 2014.

[19] R Core Team (2014). R: A language and environment for statistical computing. R Foundation for Statistical Computing, Vienna, Austria.

[20] Wilson, D., & Weber, L. (2002). Surveillance, risk and preemption on the Australian border. Surveillance & Society, 5(2): 124-141.

[21] World Health Organization (2014) WHO: Ebola Response Roadmap Situation Report -- 3 December 2014. Online report, accessed December 5.

[22] World Health Organization (2014) Data on new cases per epi week for Sierra Leone, Data published on 03 December 2014. Accessed online December 5.

